# Supplementary material for: Exploring the Successions in Microbial Community and Flavor of Daqu during Fermentation Produced by Different Pressing Patterns
Source: Foods. 2023 Jul 5;12(13):2603. doi: 10.3390/foods12132603 (PMC10340368; doi:10.3390/foods12132603)
Supplement: Supplementary file 1 [file foods-12-02603-s001.zip › foods-2475594-SI.pdf]

Figure S1. Dynamics of physicochemical and enzymatic characteristics during the fermentation of *daqu*. (A) water content, (B) acidity, (C) fermenting power, (D) saccharifying power, (E) esterifying power, (F) liquefying power. Each data point is the mean of three measurements.

Figure S2. Rarefaction curve for bacteria (A) and fungi (B) in two types of *daqu* samples based on the observed species.

Figure S3. Shared and unique microbial taxa across the two types of *daqu*. Venn diagram of genus distribution based on bacteria (A) and fungi (B) in the two types of *daqu* during fermentation process. Venn networks (C) show the shared and unique genera in the two *daqu*.

Figure S4. The principal coordinate analysis (PCoA) of the physicochemical properties in two types of *daqu* during fermentation

Table S1. Main flavor substances detected in *daqu* during fermentation

Volatile compounds	Retention time (min)	Average concentration (μg/g dry weight)										
		D0	A5	A10	A15	A25	A40	M5	M10	M15	M20	M30
**Ketones (7)**												
2-Octanone	12.673	0.346	0.000	1.367	0.072	0.000	0.000	0.000	0.058	0.000	0.000	0.000
2-Nonanone	16.348	0.000	0.000	0.075	0.000	0.138	0.000	0.057	0.000	0.000	0.000	0.000
2-Undecanone	22.763	0.000	0.000	0.034	0.000	0.000	0.000	0.046	0.000	0.000	0.000	0.000
2-Dodecanone	22.773	0.021	0.000	0.000	0.000	0.000	0.000	0.000	0.000	0.000	0.000	0.000
5,9-Undecadien-2-one, 6,10-dimethyl-, (Z)-	27.113	0.000	0.027	0.039	0.000	0.090	0.132	0.030	0.033	0.000	0.000	0.049
4-Octanone	28.41	0.000	0.000	0.000	0.000	0.023	0.000	0.000	0.000	0.055	0.014	0.000
2-Pentadecanone, 6,10,14-trimethyl-	36.585	0.000	0.011	0.000	0.000	0.014	0.000	0.018	0.000	0.000	0.000	0.000
∑(Concentration)		0.366	0.038	1.515	0.072	0.250	0.132	0.103	0.090	0.055	0.014	0.049
Σ (Type)		2	2	4	1	4	1	4	2	1	1	1
**Esters (59)**												
Hexanoic acid, methyl ester	10.244	0.047	0.058	0.087	0.000	6.107	2.720	0.000	0.000	0.000	4.242	2.591
Hexanoic acid, ethyl ester	13.105	0.032	0.222	0.000	0.262	1.311	1.996	0.621	0.167	0.188	0.610	2.197
Heptanoic acid, methyl ester	13.999	0.000	0.035	0.000	0.000	2.318	0.758	0.000	0.000	0.000	0.706	0.749
Carbonic acid, nonyl prop-1-en-2-yl ester	16.365	0.000	0.020	0.000	0.000	0.000	0.000	0.000	0.076	0.000	0.000	0.204
Benzoic acid, methyl ester	16.47	0.092	0.000	0.000	0.000	0.000	0.000	0.000	0.000	0.000	0.000	0.000
Hexanoic acid, propyl ester	16.485	0.000	0.000	0.000	0.000	0.036	0.103	0.000	0.000	0.000	0.000	0.150
Octanoic acid, methyl ester	17.473	0.039	0.260	0.161	0.040	3.154	1.909	0.041	0.057	0.029	1.262	1.383
Hexanoic acid,2-methylpropyl ester	17.600	0.000	0.000	0.000	0.000	0.085	0.023	0.000	0.000	0.000	0.054	0.000
Benzeneacetic acid, methyl ester	19.163	0.058	0.000	0.089	0.000	0.000	0.000	0.000	0.456	0.000	0.058	0.000

4-Octenoic acid, ethyl ester, (Z)-	19.55	0.014	0.000	0.000	0.000	0.000	0.000	0.020	0.000	0.000	0.000	0.000
Hexanoic acid, butyl ester	19.662	0.000	0.000	0.000	0.000	0.491	0.615	0.000	0.000	0.000	0.250	0.523
Pentadecanoic acid, 2,6,10,14-tetramethyl-, methyl ester	19.672	0.000	0.000	0.028	0.000	0.000	0.494	0.111	0.000	0.000	0.000	0.000
Octanoic acid, ethyl ester	19.828	0.000	0.078	0.000	0.018	0.171	0.000	0.000	0.021	0.000	0.097	0.481
hexanoate acid isopentyl ester	20.389	0.000	0.000	0.000	0.000	0.291	0.199	0.000	0.000	0.000	0.000	0.196
Nonanoic acid, methyl ester	20.665	0.029	0.066	0.057	0.012	0.189	0.102	0.022	0.018	0.000	0.108	0.110
Benzeneacetic acid, ethyl ester	21.261	0.000	0.021	0.000	0.000	0.053	0.058	0.000	0.000	0.000	0.000	0.048
Acetic acid, 2-phenylethyl ester	21.621	0.000	0.020	0.000	0.019	0.000	0.000	0.025	0.000	0.000	0.000	0.000
2-Nonenoic acid,2-methyl-,methyl ester	21.907	0.000	0.000	0.308	0.000	0.000	0.000	0.000	0.000	0.000	0.000	0.000
Heptanoic acid, butyl ester	22.659	0.000	0.000	0.000	0.000	0.000	0.000	0.000	0.000	0.000	0.000	0.121
Docosanoic acid, ethyl ester	22.838	0.000	0.000	0.000	0.000	0.044	0.070	0.000	0.000	0.000	0.000	0.000
Octadecanoic acid, ethyl ester	22.865	0.000	0.000	0.000	0.000	0.000	0.000	0.000	0.000	0.000	0.000	0.026
Hexanoic acid, 4-octyl ester	23.164	0.000	0.000	0.000	0.000	0.057	0.000	0.000	0.000	0.000	0.000	0.000
4-Decenoic acid, methyl ester, Z-	23.204	0.000	0.026	0.034	0.000	0.018	0.000	0.197	0.000	0.000	0.016	0.000
Carbonic acid, undecyl vinyl ester	23.44	0.000	0.000	0.016	0.000	0.000	0.000	0.027	0.000	0.000	0.027	0.016
Decanoic acid, methyl ester	23.654	0.021	0.077	0.115	0.013	0.186	0.126	0.134	0.028	0.028	0.107	0.073
4-Decenoic acid, ethyl ester, (Z)-	25.223	0.000	0.216	0.041	0.000	0.000	0.000	0.529	0.034	0.000	0.000	0.000
Hexanoic acid, hexyl ester	25.422	0.000	0.000	0.000	0.000	0.324	0.879	0.000	0.000	0.000	0.043	0.513
Decanoic acid, ethyl ester	25.671	0.000	0.041	0.000	0.000	0.000	0.046	0.075	0.000	0.000	0.000	0.024
hydrate acid 7-epi-trans-sesquisabinene ester	26.713	0.000	0.000	0.000	0.017	0.021	0.000	0.000	0.000	0.000	0.034	0.000
acetate acid Linalyl ester	27.375	0.000	0.000	0.000	0.000	0.000	0.026	0.000	0.000	0.000	0.000	0.000
6-Octadecenoic acid, methyl ester, (Z)-	28.7	0.000	0.000	0.000	0.000	0.062	0.000	0.000	0.033	0.000	0.000	0.062
Dodecanoic acid, methyl ester	29.09	0.025	0.088	0.169	0.028	0.213	0.157	0.068	0.041	0.071	0.125	0.077
Glutaric acid, (2-methylcyclohex-1-enyl)methyl tridec-2-yn-1-yl ester	29.178	0.000	0.000	0.000	0.000	0.000	0.000	0.297	0.000	0.000	0.000	0.000

9-hexadecenoate Ethyl ester	30.439	0.000	0.011	0.000	0.000	0.000	0.000	0.034	0.000	0.000	0.000	0.000
diisobutyrate acid 2,2,4-Trimethyl-1,3-pentanediol ester	30.7	0.000	0.000	0.021	0.022	0.000	0.022	0.000	0.000	0.031	0.000	0.030
Pentanoic acid, 2,2,4-trimethyl-3-carboxyisopropyl, isobutyl ester	30.701	0.000	0.021	0.000	0.000	0.000	0.000	0.000	0.000	0.000	0.000	0.000
Tridecanoic acid, 12-methyl-, methyl ester	33.108	0.000	0.179	0.000	0.000	0.000	0.000	0.093	0.035	0.000	0.000	0.000
Cyclopentaneundecanoic acid, methyl ester	33.116	0.000	0.000	0.000	0.000	0.000	0.030	0.000	0.000	0.000	0.000	0.000
13-Tetradecynoic acid, methyl ester	33.122	0.000	0.016	0.000	0.000	0.000	0.029	0.000	0.000	0.000	0.000	0.000
13,16-Octadecadiynoic acid, methyl ester	33.142	0.000	0.000	0.000	0.000	0.047	0.000	0.000	0.035	0.052	0.000	0.025
5-Octadecenoic acid, methyl ester	33.336	0.000	0.024	0.105	0.010	0.053	0.029	0.000	0.000	0.000	0.063	0.015
tetradecanoate Methyl ester	33.977	0.152	0.358	1.001	0.183	0.761	0.510	0.479	0.000	0.327	1.028	0.255
Pentadecanoic acid, 14-methyl-, methyl ester	35.401	0.000	0.027	0.207	0.000	0.063	0.038	0.085	0.033	0.023	0.000	0.021
Tetradecanoic acid, 12-methyl-, methyl ester, (S)-	35.579	0.000	0.066	0.188	0.014	0.000	0.000	0.172	0.083	0.044	0.000	0.052
13-Tetradecynoic acid, methyl ester	35.623	0.000	0.000	0.000	0.000	0.173	0.000	0.000	0.000	0.000	0.050	0.000
Pentadecanoic acid, methyl ester	36.228	0.051	0.136	0.546	0.056	0.336	0.186	0.184	0.116	0.190	0.224	0.111
Pentadecanoic acid, isopropyl ester	36.696	0.000	0.027	0.000	0.000	0.000	0.000	0.000	0.000	0.000	0.000	0.038
7,10-Hexadecadienoic acid, methyl ester	37.669	0.000	0.000	0.000	0.000	0.000	0.000	0.000	0.000	0.028	0.124	0.000
9,12-Hexadecadienoic acid, methyl ester	37.674	0.000	0.000	0.000	0.000	0.000	0.000	0.000	0.000	0.000	0.034	0.000
7-Hexadecenoic acid, methyl ester, (Z)-	37.821	0.038	0.084	0.494	0.041	0.242	0.100	0.228	0.086	0.211	0.000	0.094
Hexadecanoic acid, methyl ester	38.412	1.397	3.393	8.501	1.453	8.188	6.284	4.510	3.974	5.436	6.441	4.977
Hexadecanoic acid, ethyl ester	39.773	0.000	0.151	0.042	0.028	0.087	0.106	0.060	0.022	0.000	0.023	0.020
Heptadecanoic acid, methyl ester	39.85	0.000	0.000	0.063	0.000	0.028	0.032	0.056	0.015	0.000	0.038	0.000
Cyclopropaneoctanoic acid, 2-hexyl-, methyl ester	39.941	0.000	0.000	0.079	0.000	0.045	0.000	0.000	0.028	0.000	0.027	0.000

9,12-Octadecadienoic acid, methyl ester	41.797	0.142	0.595	1.083	0.080	1.962	1.049	0.188	0.328	0.609	1.358	0.973
9-Octadecenoic acid (Z)-, methyl ester	41.948	0.253	0.582	2.405	0.155	1.862	1.103	0.675	0.592	1.001	0.922	0.915
stearate acid Methyl ester	42.536	0.000	0.022	0.104	0.013	0.063	0.059	0.049	0.019	0.049	0.053	0.043
9,11-Octadecadienoic acid, methyl ester, (E,E)-	42.994	0.000	0.013	0.050	0.000	0.145	0.000	0.000	0.000	0.000	0.157	0.033
11,14-Eicosadienoic acid, methyl ester	43.115	0.000	0.000	0.000	0.000	0.000	0.000	0.000	0.000	0.000	0.017	0.000
9,12-Octadecadienoic acid, methyl ester, (E,E)-	43.198	0.000	0.000	0.027	0.000	0.054	0.000	0.000	0.000	0.000	0.043	0.096
Σ(Concentration)		2.390	6.751	16.199	2.453	29.239	19.859	8.978	6.329	8.314	18.355	17.238
Σ(Type)		15	30	28	18	35	31	26	24	26	32	35
**Aldehydes (8)**												
Benzaldehyde	11.662	0.000	0.000	0.000	0.000	0.000	0.000	0.855	0.142	0.000	0.000	0.000
Benzeneacetaldehyde	14.726	0.067	0.233	0.000	0.031	0.000	0.000	0.186	0.000	0.020	0.000	0.109
2-Octenal, (E)-	15.254	0.024	0.026	0.000	0.000	0.000	0.000	0.000	0.000	0.000	0.000	0.000
Nonanal	16.832	0.156	0.071	0.043	0.048	0.105	0.147	0.086	0.037	0.025	0.070	0.106
2-Phenylpropenal	18.506	0.000	0.082	0.154	0.000	0.000	0.000	0.163	0.078	0.078	0.000	0.000
2-Nonenal, (E)-	18.682	0.059	0.039	0.000	0.000	0.000	0.000	0.026	0.000	0.000	0.000	0.017
Decanal	20.15	0.016	0.000	0.011	0.024	0.054	0.078	0.000	0.011	0.000	0.022	0.000
2-Tridecenal, (E)-	28.301	0.000	0.000	0.000	0.045	0.000	0.000	0.000	0.000	0.000	0.000	0.000
Σ(Concentration)		0.322	0.451	0.208	0.148	0.158	0.225	1.315	0.037	0.123	0.092	0.232
Σ(Type)	8	5	5	3	4	2	2	5	4	3	2	3
**Alcohols (16)**												
2,3-Butanediol	5.225	0.000	0.000	0.327	0.000	0.000	0.000	0.691	0.327	0.000	0.000	2.849
2,3-Butanediol, [R-(R*,R*)]-	5.55	0.000	0.000	0.000	0.000	0.000	0.000	1.624	0.000	0.000	0.000	3.758
4-Ethylcyclohexanol	12.752	0.000	0.428	0.000	0.000	0.000	0.000	0.000	0.000	0.000	0.000	0.000
Phenylethyl Alcohol	17.128	0.000	0.000	0.000	0.000	0.000	0.000	0.135	0.000	0.000	0.000	0.000

1-Heptanol, 2-propyl-	21.897	0.000	0.033	0.000	0.000	0.000	0.000	0.000	0.000	0.000	0.000	0.000
2-Ethyl-1-dodecanol	21.926	0.000	0.000	0.000	0.000	0.000	0.000	0.000	0.000	0.000	0.000	0.057
2-Methoxy-4-vinylphenol	23.345	0.000	0.024	0.000	0.000	0.000	0.000	0.000	0.000	0.000	0.000	0.000
1-Octanol, 2-butyl-	23.45	0.000	0.037	0.000	0.014	0.000	0.000	0.000	0.018	0.000	0.000	0.000
1H-Cycloprop[e]azulen-4-ol, decahydro-1,1,4,7-tetramethyl-,[1aR-(1a.alpha.,4.beta. ,4a.beta.,7.alpha.,7a.beta.,7b.alpha.)]-	24.076	0.000	0.013	0.000	0.000	0.000	0.000	0.000	0.000	0.000	0.000	0.000
1-Dodecanol	25.624	0.000	0.000	0.000	0.000	0.000	0.000	0.000	0.040	0.000	0.000	0.022
(1S,2R,5R)-2-Methyl-5-((R)-6-methylhept-5-en-2-yl)bicyclo[3.1.0]hexan-2-ol	26.728	0.000	0.000	0.000	0.000	0.000	0.000	0.000	0.028	0.000	0.000	0.000
11-Tridecyn-1-ol	27.924	0.000	0.000	0.000	0.000	0.000	0.000	0.000	0.000	0.000	0.037	0.000
3-Tetradecyn-1-ol	28.307	0.000	0.000	0.000	0.000	0.064	0.000	0.000	0.000	0.000	0.000	0.000
trans-2-Dodecen-1-ol	32.12	0.000	0.000	0.000	0.000	0.000	0.089	0.000	0.000	0.000	0.000	0.000
2-Tridecen-1-ol, (E)-	32.141	0.000	0.000	0.000	0.000	0.086	0.000	0.000	0.000	0.000	0.000	0.000
2-Naphthalenemethanol, decahydro-.alpha.,.alpha.,4a-trimethyl-8-methylene-, [2R-(2.alpha.,4a.alpha.,8a.beta.)]	32.918	0.000	0.030	0.058	0.000	0.000	0.000	0.000	0.000	0.000	0.000	0.000
Σ (Concentration)		0.000	0.565	0.384	0.014	0.151	0.089	2.449	0.412	0.000	0.037	6.686
Σ (Type)		0	6	2	1	2	1	3	4	0	1	4
**Acids (4)**												
Hexanoic acid	13.67	0.000	0.000	0.000	0.000	0.103	0.461	0.000	0.000	0.000	0.000	0.000
n-Decanoic acid	16.381	0.000	0.000	0.107	0.000	0.000	0.000	0.000	0.000	0.000	0.000	0.000
8,11,14-Eicosatrienoic acid, (Z,Z,Z)-	27.905	0.000	0.000	0.000	0.000	0.000	0.000	0.021	0.000	0.000	0.000	0.000
Linoelaidic acid	43.353	0.000	0.000	0.000	0.000	0.047	0.000	0.000	0.000	0.000	0.000	0.000
Σ (Concentration)		0.000	0.000	0.107	0.000	0.150	0.461	0.021	0.000	0.000	0.000	0.000
Σ (Type)		0	0	1	0	2	1	1	0	0	0	0

**Aromatics (1)**												
Phenylethyl Alcohol	17.128	0.000	0.000	0.000	0.000	0.000	0.000	0.135	0.000	0.000	0.000	0.000
∑(Concentration)		0.000	0.000	0.000	0.000	0.000	0.000	0.135	0.000	0.000	0.000	0.000
∑(Type)		0	0	0	0	0	0	1	0	0	0	0
**Phenols (1)**												
2-Methoxy-4-vinylphenol	23.345	0.000	0.024	0.000	0.000	0.000	0.000	0.000	0.000	0.000	0.000	0.000
∑(Concentration)		0.000	0.024	0.000	0.000	0.000	0.000	0.000	0.000	0.000	0.000	0.000
∑(Type)		0	1	0	0	0	0	0	0	0	0	0
**Pyrazine (1)**												
Pyrazine, tetramethyl-	16.197	0.000	0.000	0.112	0.075	0.161	0.000	0.000	0.000	0.054	0.000	0.000
∑(Concentration)		0.000	0.000	0.112	0.075	0.161	0.000	0.000	0.000	0.054	0.000	0.079
∑(Type)		0	0	1	1	1	0	0	0	1	0	0
**Others (2)**												
Bi-1-cycloocten-1-yl	29.196	0.133	0.000	0.000	0.092	0.000	0.000	0.080	0.000	0.000	0.000	0.000
N-(Trifluoroacetyl)-N,O,O',O''-tetrakis(trimethylsilyl)norepinephrine	31.60	0.032	0.101	0.210	0.000	0.000	0.092	0.000	0.000	0.000	0.079	0.174
∑(Concentration)		0.166	0.101	0.210	0.092	0.000	0.092	0.080	0.000	0.000	0.079	0.174
∑(Type)		2	1	1	1	0	1	1	0	0	1	1
**Σ (Concentration) in total**		3.244	8.042	18.699	2.939	29.949	20.858	13.080	7.145	8.491	18.654	24.379
**Σ (Type) in total**		24	45	40	26	46	37	42	37	31	37	44

Table S2. High quality sequence based on 16S rRNA and ITS sequencing

No.	No. of sequences				Proportion (%)	
	Effective of sequence		High-quality sequences			
	Bac.	Fun.	Bac.	Fun.	Bac.	Fun.
D0	59381	51598	57663	51259	97.11	99.34
A5	47875	90493	47033	90377	98.24	99.87
A10	32871	50143	32331	50102	98.36	99.92
A15	37098	56980	35975	56113	96.97	98.48
A25	87201	62503	85573	62077	98.13	99.32
A40	59253	45786	57566	45126	97.15	98.56
M5	59817	55121	55275	54004	92.41	97.97
M10	58699	42936	55588	42495	94.70	98.97
M15	57476	85714	56189	84198	97.76	98.23
M20	59995	78827	57908	76782	96.52	97.41
M30	60445	84471	55980	74965	92.61	88.75
Average	59381	51598	57663	51259	89.48	86.32

Table S3. Difference of α-diversity indices for microbial communities based on 16S rRNA and ITS sequencing

No.	Observed OTUs		Abundance index				Diversity index			
			ACE		Chao1		Simpson		Shannon	
	Bac.	Fun.	Bac.	Fun.	Bac.	Fun.	Bac.	Fun.	Bac.	Fun.
D0	375	222	402	250.990	520.71	249.660	0.250	0.240	2.480	2.450
A5	259	79	233.470	87.850	244.08	93.810	0.590	0.075	3.140	2.990
A10	266.500	74.500	300.260	78	256.09	78.100	0.290	0.140	3.190	2.600
A15	329	90	343.130	99.660	286.145	111.720	0.050	0.140	3.880	2.440
A25	311.500	123.500	331.2400	150.120	339.710	141.670	0.063	0.071	3.960	3.051
A40	201	89	268.370	116.380	257.390	117.630	0.110	0.700	2.820	0.860
M5	79.330	139	108.280	250.990	101.880	249.660	0.130	0.240	2.420	2.450
M10	88.500	43.500	199.420	126.080	153.870	127.720	0.180	0.200	2.300	2.180
M15	175	96	176.170	91.130	175.760	59.560	0.100	0.910	2.500	0.230
M20	217	48.500	297.180	117.940	280.280	112.810	0.054	0.320	3.320	1.560
M30	137.330	73.500	236.470	80.360	205.270	66.300	0.250	0.340	2.210	1.150

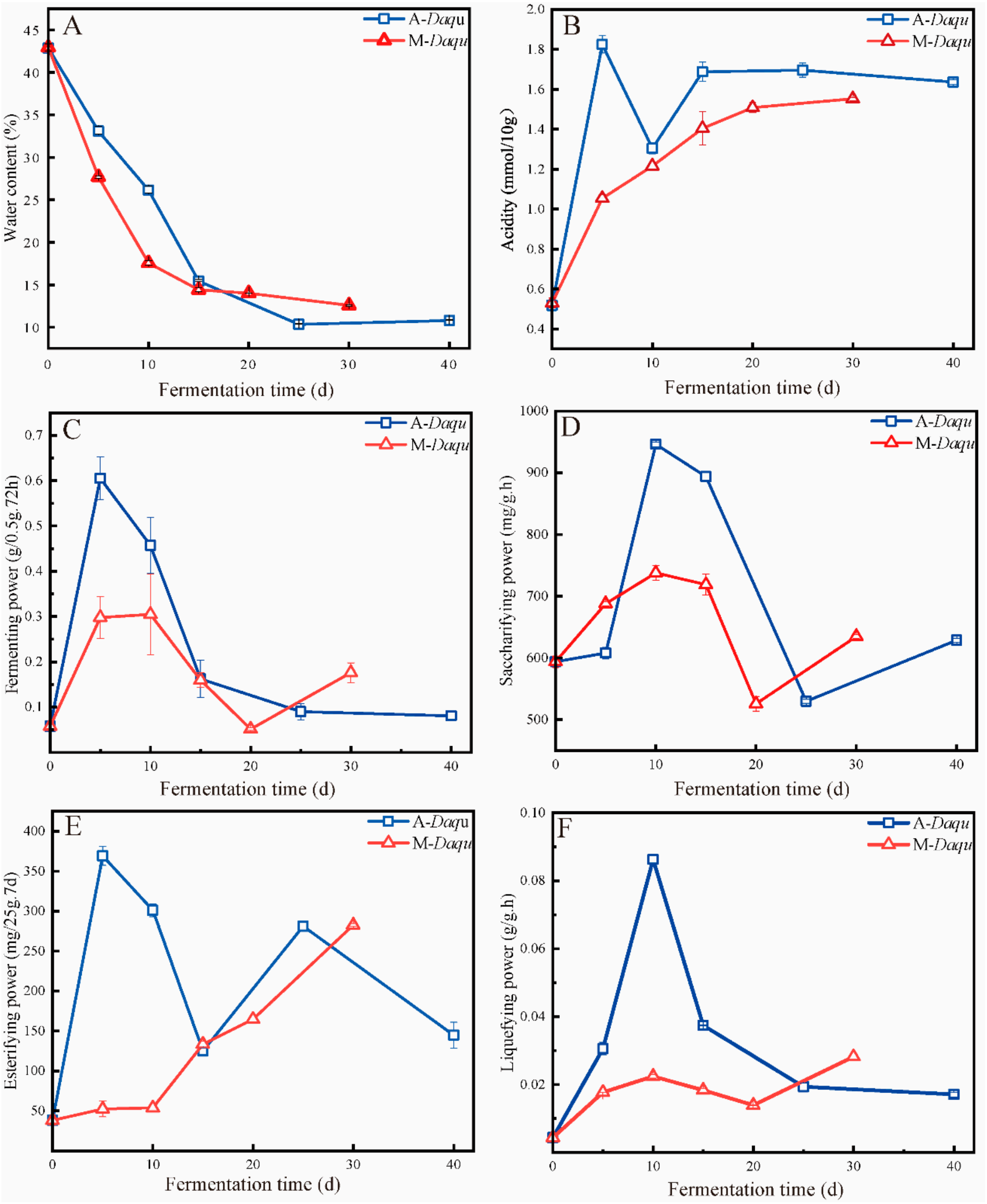
A
B
C
D
E
F
A-Daqu
M-Daqu
Water content (%)
Acidity (mmol/10g)
Fermenting power (g/0.5g.72h)
Saccharifying power (mg/g.h)
Esterifying power (mg/25g.7d)
Liquefying power (g/g.h)
Fermentation time (d)

Figure S1

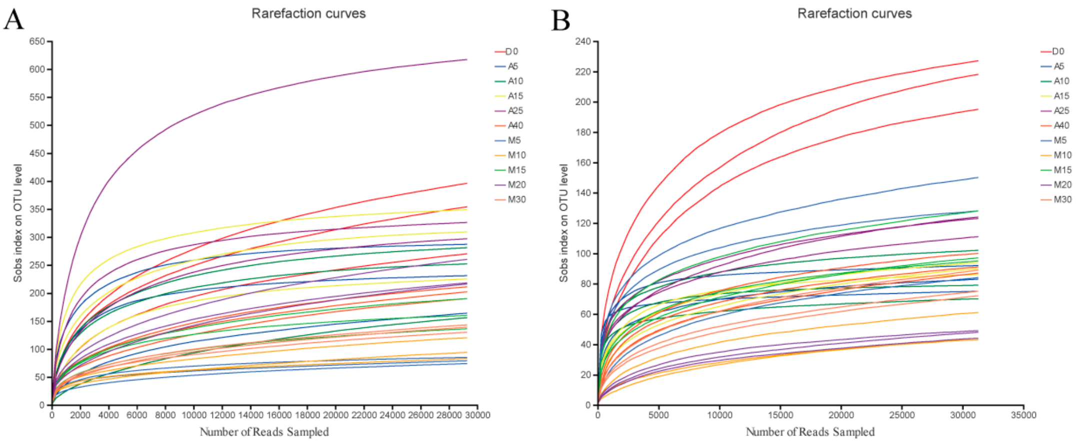
A
Rarefaction curves
Sobs index on OTU level
Number of Reads Sampled
D0
A5
A10
A15
A25
A40
M5
M10
M15
M20
M30
B
Rarefaction curves
Sobs index on OTU level
Number of Reads Sampled
D0
A5
A10
A15
A25
A40
M5
M10
M15
M20
M30

Figure S2

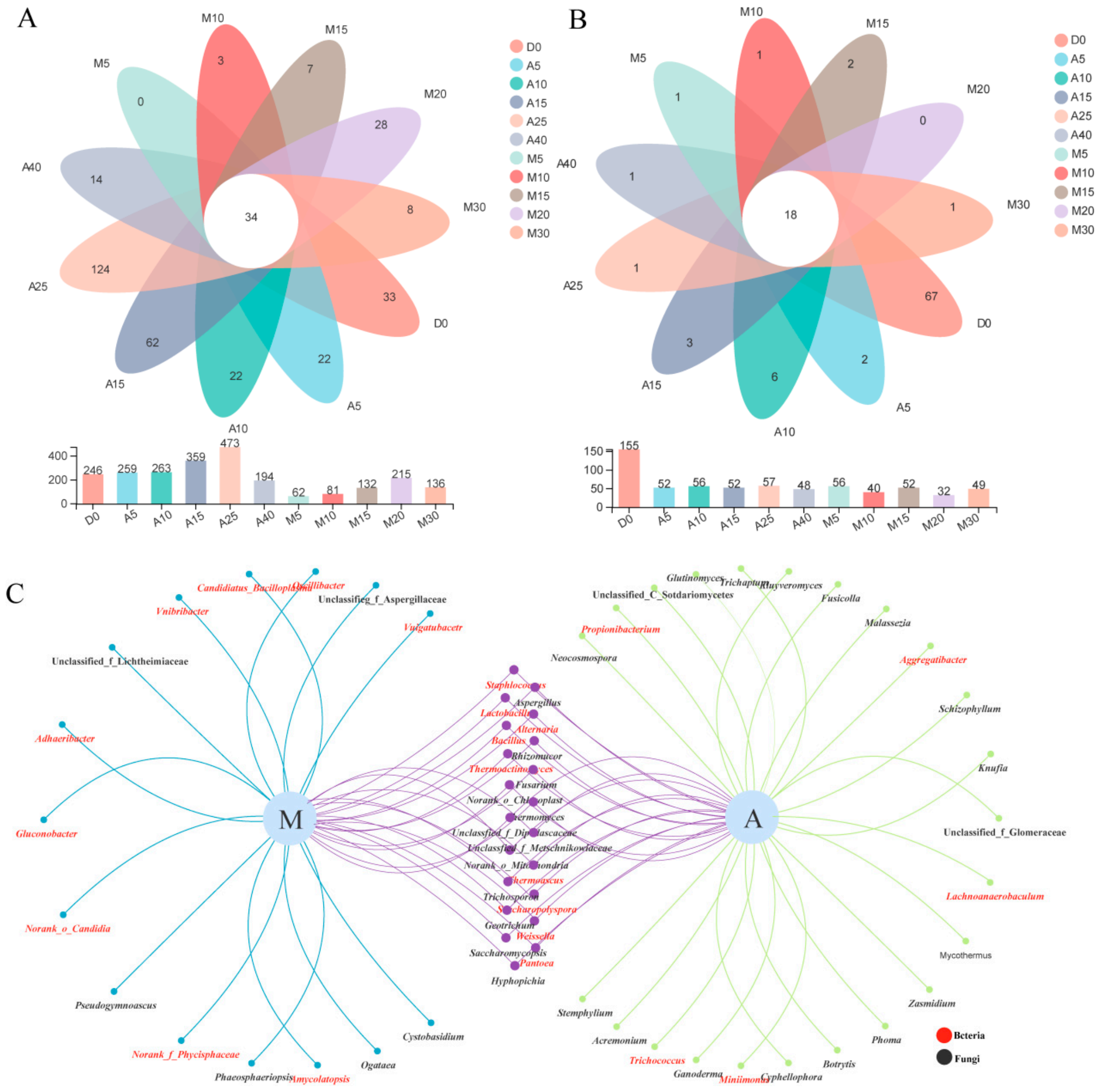
A
B
C
D0
A5
A10
A15
A25
A40
M5
M10
M15
M20
M30
M
A
Bcteria
Fungi
Unclassified_f_Aspergillaceae
Unclassified_f_Lichtheimiaceae
Vnibribacter
Vulgatubacetr
Adhaeribacter
Gluconobacter
Norank_o_Candidia
Pseudogymnoascus
Norank_f_Phycisphaceae
Phaeosphaeriopsis
Amycolatopsis
Ogataea
Cystobasidium
Unclassified_C_Sotdariomycetes
Glutinomyces
Fusicolla
Malassezia
Propionibacterium
Neocosmospora
Aggregatibacter
Schizophyllum
Knufia
Unclassified_f_Glomeraceae
Lachnoanaerobaculum
Mycothermus
Zasmidium
Phoma
Botrytis
Ganoderma
Trichococcus
Acremonium
Stemphylium
Aspergillus
Alternaria
Rhizomucor
Fusarium
Trichosporon
Geotrichum
Saccharomycopsis
Pantoea
Hyphopichia

Figure S3

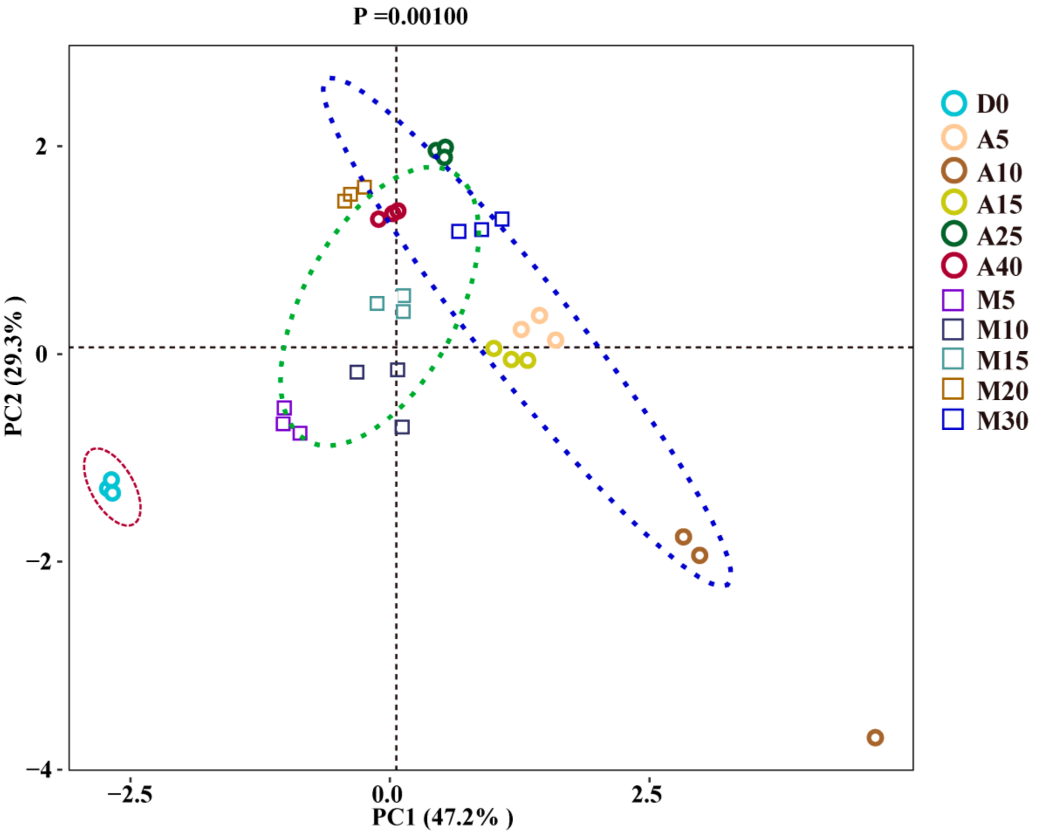
P =0.00100
PC2 (29.3%)
PC1 (47.2%)
2
0
−2
−4
−2.5
0.0
2.5
D0
A5
A10
A15
A25
A40
M5
M10
M15
M20
M30

Figure S4
